# Heart as white as stone, reborn: an unusual case report of idiopathic myocardial calcifications leading to end-stage heart failure and cardiac transplantation

**DOI:** 10.1093/ehjcr/ytaf523

**Published:** 2025-10-08

**Authors:** Alexander Fardman, Yael Peled, Orly Goitein, Shira Goldenberg, Roy Beigel

**Affiliations:** Department of Cardiology, The Heart Center, Sheba Medical Center, Tel Hashomer, Ramat-Gan 5262000, Israel; Department of Cardiology, The Heart Center, Sheba Medical Center, Tel Hashomer, Ramat-Gan 5262000, Israel; Diagnostic Radiology, Sheba Medical Center, Tel Hashomer, Ramat-Gan 5262000, Israel; Department of Family Medicine, Maccabi Health Services, Ahuza Street, Raanana 4345020, Israel; Department of Cardiology, The Heart Center, Sheba Medical Center, Tel Hashomer, Ramat-Gan 5262000, Israel

**Keywords:** Myocardial calcifications, Heart failure, Heart transplantation, Case report

## Abstract

**Background:**

Massive myocardial calcifications without an underlying cause are a rare entity of unknown pathophysiology that could result in heart failure with preserved ejection fraction and restrictive physiology. The role of heart transplantation in these cases is unclear due to a lack of data and a concern that calcifications could reoccur in a transplanted organ.

**Case summary:**

We describe herein a 61-year-old patient who presented with new-onset heart failure with preserved ejection fraction. Multimodality imaging evaluation was consistent with restrictive cardiomyopathy secondary to massive myocardial calcifications. Extensive workup did not detect an underlying aetiology that could lead to development of myocardial calcifications. Eventually, at the age of 66, the patient underwent successful heart transplantation without evidence of calcification recurrence during a long-term follow-up of 9 years.

**Conclusion:**

Massive myocardial calcifications could result in end-stage heart failure. Heart transplantation might be considered a safe and reasonable therapeutic option in patients with idiopathic myocardial calcifications.

Learning points

- Reports on massive myocardial calcification are scarce, and until recently, this entity was usually found only at post-mortem autopsies and explained by either dystrophic or metastatic mechanisms.
- Massive myocardial calcifications could lead to heart failure with preserved ejection fraction and restrictive physiology.
- Heart transplantation is safe and feasible in patients with myocardial calcifications without an apparent aetiology.

## Introduction

Reports on massive myocardial calcification are scarce, and until recently, this entity was usually found only at autopsy. Myocardial calcifications are thought to occur primarily by two potential mechanisms: either dystrophic or metastatic.^1^ Dystrophic calcifications have been reported to occur due to calcium deposition in necrotic tissue such as secondary to a myocardial insult, mainly due to a prior infarction^2,3^ or due to other, less common, causes such as a cardiac haematoma^4^ and endomyocardial fibrosis.^5^ Metastatic deposition of calcium has been reported secondary to conditions such as renal failure^6^ and hyperparathyroidism.^7^ Extensive epipericardial and intramyocardial calcification secondary to massive mitral annular calcification has been reported but these rarely occur.^8^ It is uncommon for myocardial calcification to be found in individuals without associated conditions causing metastatic deposition or a clinical history of myocardial damage, with very few case reports of myocardial calcifications occurring without any apparent aetiology previously published.^9,10^

Treatment options for patients with extensive myocardial calcifications who develop heart failure are limited, and whether or not these patients could be heart transplant candidates is unknown. We present a case of a patient with massive myocardial calcifications that eventually led to intractable heart failure and who underwent successful heart transplantation.

## Summary figure

**Figure:**
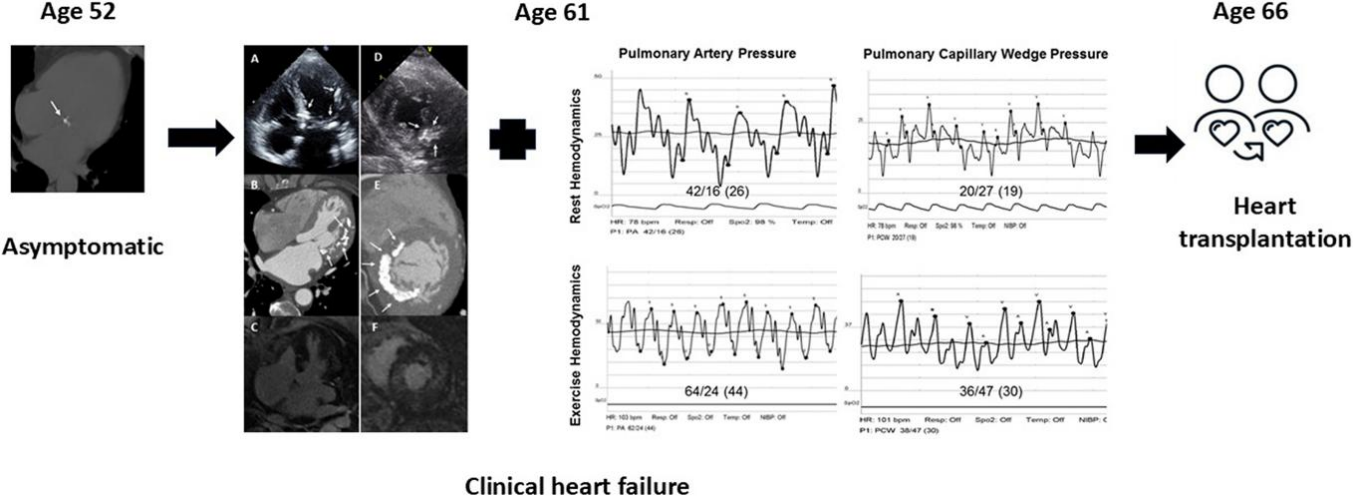


## Case presentation

**Figure 1:**
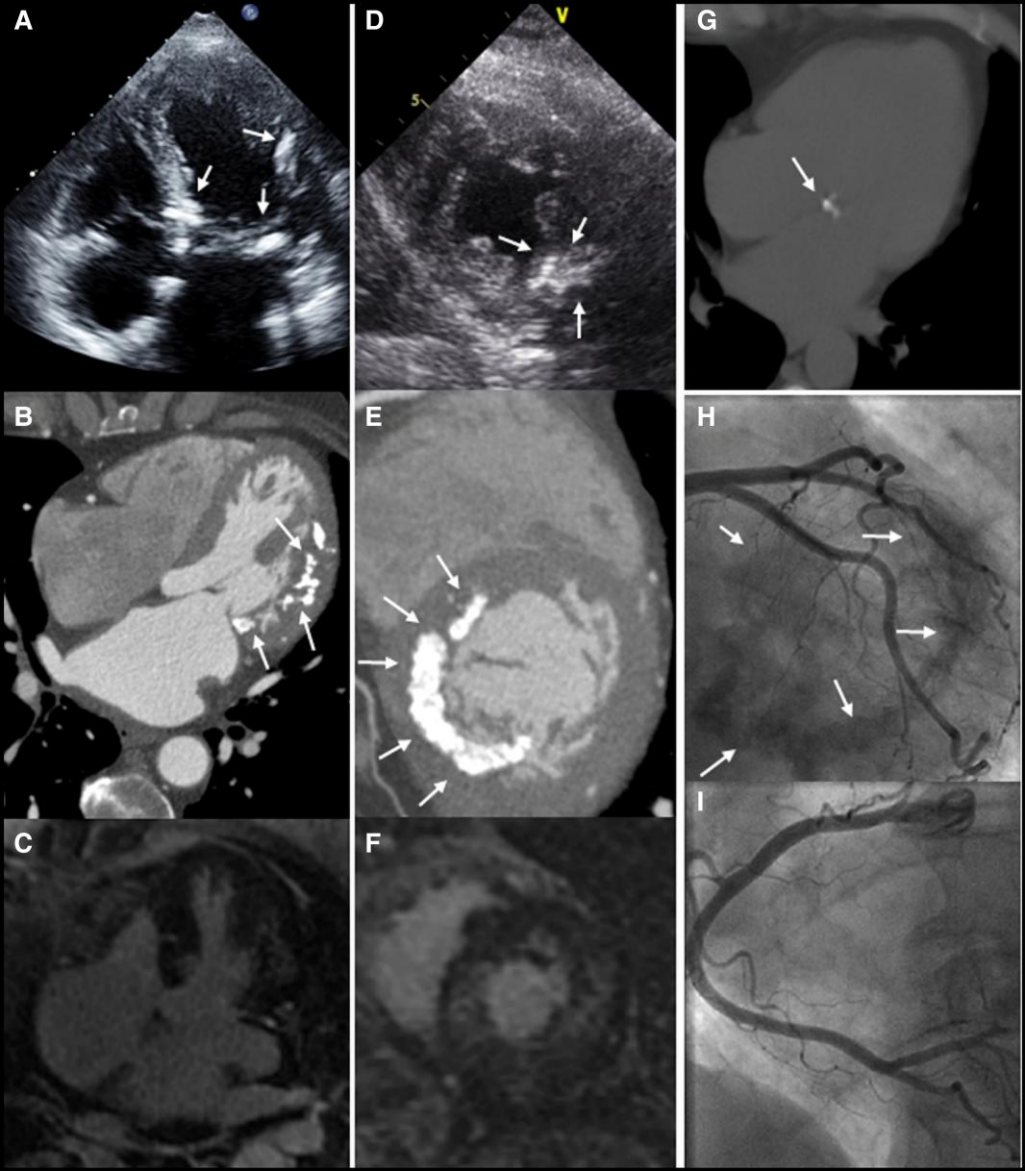
Different imaging modalities demonstrating extensive myocardial calcifications. Four-chamber view of the (*A*) echocardiographic, (*B*) cardiac computed tomographic angiography, and (*C*) cardiac magnetic resonance imaging studies of Patient 1 demonstrating gross mitral annular and myocardial calcifications (arrows) along with diffuse, patchy, mid-myocardial delayed enhancement involving the lateral wall. The corresponding short-axis views of the (*D*) echocardiographic, (*E*) cardiac computed tomographic angiography, and (*F*) cardiac magnetic resonance imaging studies also demonstrate gross myocardial calcifications (arrows) along with diffuse, patchy, mid-myocardial delayed enhancement involving the anterolateral wall. (*G*) Chest computed tomography obtained from the same patient 9 years prior to the present presentation which shows only minimal mitral annular calcification with no myocardial involvement. (*H*, *I*) Coronary angiography demonstrating evidence of severe myocardial calcifications (arrows) and no evidence of coronary artery disease within the (*H*) left and (*I*) right coronary arteries along with.

**Figure 2:**
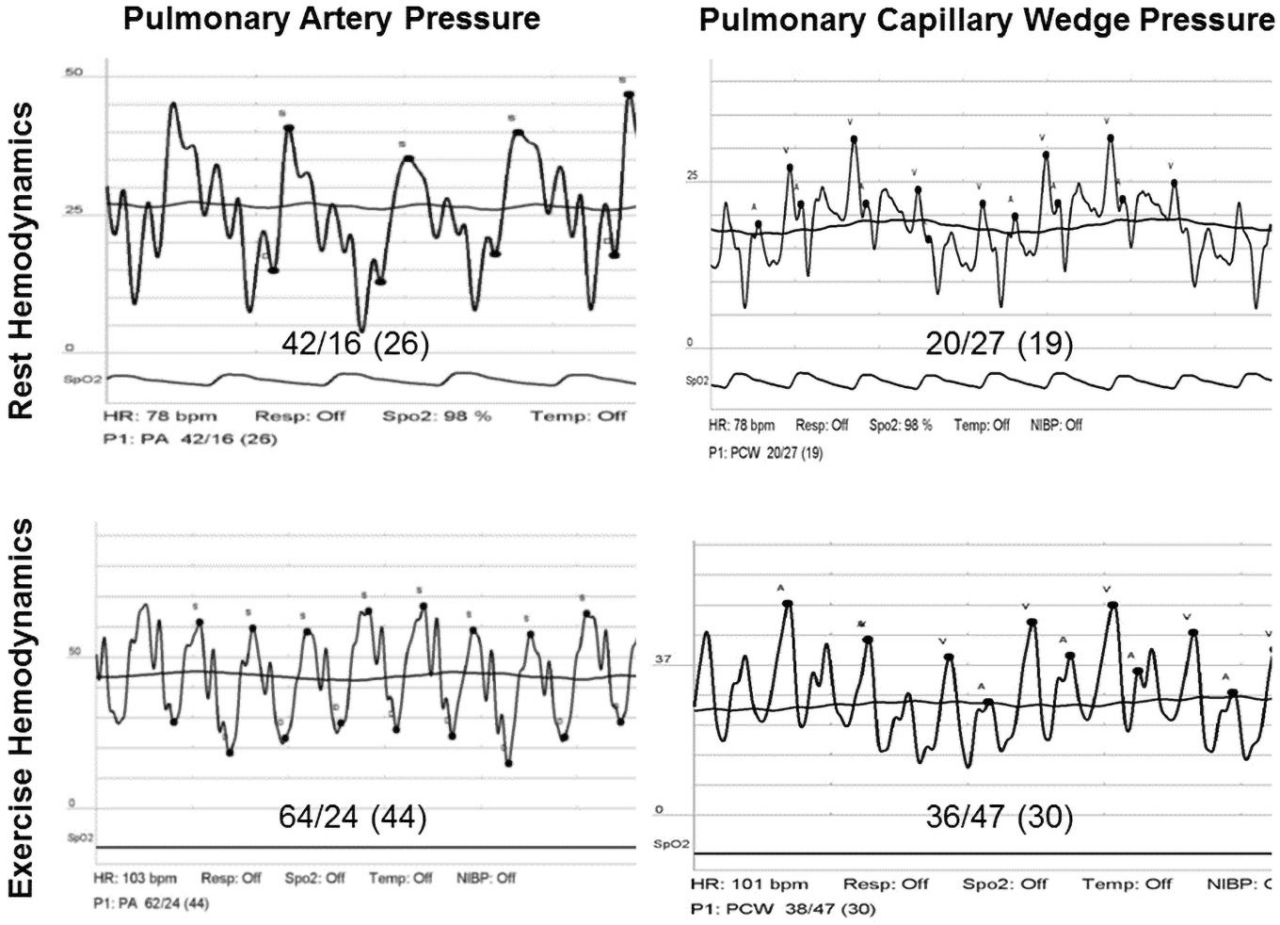
Right heart study, haemodynamic findings during rest and upon supine exercise. Tracings of the pulmonary artery pressure (left) and pulmonary capillary wedge pressure (right) during a right heart catheterization. Top: measurements taken at baseline demonstrating a very mildly elevated pulmonary artery pressure (mean 26 mmHg) and a slightly elevated pulmonary capillary wedge pressure (mean 19 mmHg). Bottom: during supine exercise, the patient became symptomatic with significant elevation in both pulmonary artery pressure (mean 44 mmHg) and pulmonary capillary wedge pressure (mean 30 mmHg).

Our patient is a 61**-**year-old female with a medical history remarkable only for hyperlipidaemia. The patient was referred for evaluation due to complaints of shortness of breath and non-specific chest pain of a few weeks’ duration. The patient denied any other systemic symptoms. The family history was unremarkable as well. Upon examination, the patients’ vital signs were normal, and the physical exam was unremarkable and non-contributory. Initial blood analysis revealed a negative troponin level, normal serum electrolytes, and a normal blood count. The electrocardiogram (ECG) showed a normal sinus rhythm of 60 beats per minute without any ST–T wave changes. A transthoracic echocardiogram (TTE) was obtained and demonstrated a normal left ventricular ejection fraction, mild mitral regurgitation (MR), and gross calcifications of the mitral annulus extending into the myocardium (*Figure 1A, D*). Echocardiographic Doppler findings were consistent with elevated left-sided filling pressures—the E/A ratio was 2.3, septal e’ was 4.6 m/s, and lateral e’ was 5.5 m/s, giving a septal E/e’ ratio of 30.6 and a lateral E/e’ ratio of 25.5 consistent with Grade 2 diastolic dysfunction. Additionally, the left atrium was enlarged with an A–P diameter of 4.6 cm, an end-systolic area of 33.6 cm^2^ an and a left atrial volume index of 71 ml/m^2^. The patient further underwent cardiac computed tomography angiography (CCTA) which demonstrated mild non-obstructive coronary artery disease. However, severe calcification of the mitral annulus, as well as significant calcifications protruding into the left ventricular myocardium, primarily involving the anterolateral wall (*Figure 1B, E*), were noticed. The CCTA scan was compared to a non-contrast chest computed tomography (CT) study obtained 9 years prior which revealed only mild mitral annular calcification without any evidence of myocardial calcifications (*Figure 1G*). Further evaluation of the current findings by cardiac magnetic resonance imaging demonstrated diffuse, patchy, mid-myocardial delayed enhancement primarily involving the anterolateral wall (*Figure 1C, F*) corresponding to the areas of calcification on CCTA. Blood tests for serum electrolytes, calcium and phosphor levels, renal function tests, and parathyroid hormone levels were all within normal limits. There were no signs of calcifications in any other body organ. Due to continuing complaints of chest pain at follow-up, the patient further underwent invasive coronary angiography which did not demonstrate any evidence of coronary artery disease (*Figure 1H, I*); however, massive calcifications were again noted upon fluoroscopy (*Figure 1H*). A right heart haemodynamic study (*Figure 2*) demonstrated a resting right atrial pressure of 5 mmHg which increased to 9 mmHg following exercise manoeuvres. The resting mean pulmonary artery pressure (MPAP) was mildly elevated up to 26 mmHg with a pulmonary capillary wedge pressure (PCWP) of 19 mmHg and a left ventricular end diastolic pressure of 21 mmHg. Resting transpulmonary gradient (TPG) was 7, and the diastolic pulmonary gradient (DPG) was 3, supporting the presence of post-capillary pulmonary hypertension. Following supine exercise, MPAP and PCWP increased to 44 and 30 mmHg, respectively. Prominent V waves up to 47 mmHg were observed during tracing of the PCWP, without echocardiographic evidence of significant MR. The cardiac index was measured using the direct Fick technique as 2.8 L/min/m^2^. The calculated pulmonary vascular resistance was 1.26 Wood units. The patient completed a cardiopulmonary exercise test (CPET) which demonstrated a reduced peak VO2 of 12.3 ml/min/kg and a significantly increased ventilation (VE) to carbon dioxide (VCO2) slope of up to 44.9.

The findings from the different imaging studies combined with haemodynamic measurements were consistent with restrictive cardiomyopathy secondary to massive myocardial calcifications causing heart failure with preserved ejection fraction, without an apparent aetiology. Therapy with beta-blockers and diuretics was initiated with mild improvement in symptoms. However, at 4 years from the initial presentation, there was a gradual increasing effort intolerance with limitation in performing simple daily activities, orthopnoea, and paroxysmal nocturnal dyspnoea despite medical therapy. It should be noticed that sodium glucose transporter inhibitors-2 (SGLT2-I) were not available at that point, and the patients’ medical therapy was consistent with current recommendations and included diuretics, blood pressure control, and beta-blockers. Despite therapy, the patient was hospitalized due to pulmonary congestion, and home treatment with oxygen supplementation was initiated. Given the severity of her heart failure symptoms under optimal therapy, recurrent hospitalizations due to pulmonary congestion, and objective evidence of exercise capacity limitation secondary to restrictive physiology combined with lack of significant comorbidities, the decision was made to pursue evaluation for heart transplantation, and eventually she was listed as a heart transplant candidate. At the age of 66, the patient underwent heart transplantation. Pathological examination of the explanted heart revealed massive myocardial calcifications of the left ventricle and the mitral valve annulus. The surrounding myocardium was characterized by myocyte hypertrophy and focal vacuolization. Focal, mild interstitial and scar-like fibrosis as well as fatty infiltration were discovered. The right ventricle consisted of hypertrophic myocytes and fatty infiltration. The tricuspid, pulmonary, and aortic valves revealed fibrosis. Immunohistochemical techniques confirmed the presence of extensive calcifications and fibrosis, and no amyloid infiltration was detected.

Currently, 9 years post-transplant, at the age of 75, the patient is doing well; she has resumed normal daily activities. Her immunosuppressive regimen consists of everolimus, mycophenolate mofetil, and low-dose corticosteroids. Routine surveillance with endomyocardial biopsies did not reveal either clinically significant episodes of rejection or recurrence of myocardial calcifications in the transplanted heart. Repeat, periodic echocardiograms and fluoroscopy did not reveal any recurrence of myocardial calcifications.

## Discussion

Cases of myocardial calcifications have been described for several decades dating back to the 1940s with initial reports based on findings from pathology examinations.^11^ With the growing use of non-invasive imaging, especially CCTA, there are increasing reports of myocardial calcifications associated with^12^ and without^13,14^ identifying a primary cause. In theory, tissue calcification can be caused due to an inflammatory process leading to calcification of the myocardial tissue. While there are no reports of myocardial calcifications due to infectious aetiologies, studies looking into murine models of viral myocarditis have shown it to induce coronary spasm leading to myocyte necrosis, fibrosis, and dystrophic calcification.^15^ However, to date, no subsequent findings have been reported to be associated with myocarditis in humans. In addition, no clinical or pathological findings supported an underlying aetiology of prior myocarditis in the current case. Our patient was found to have findings consistent with restrictive cardiomyopathy secondary to massive calcifications within the myocardium without an apparent aetiology. Restrictive cardiomyopathy consists of a heterogeneous group of diseases usually defined by the coexistence of persistent restrictive pathophysiology, diastolic dysfunction, non-dilated ventricles, and atrial dilatation, regardless of ventricular wall thickness and systolic function. A few previous cases suggested that myocardial calcifications should be added to the list of conditions causing impairment of left ventricular filling.^16–18^ Extensive myocardial calcifications surrounding the left ventricle produce an eggshell effect resulting in a restrictive non-dilated phenotype. In those patients who develop advanced heart failure symptoms despite optimal conservative therapy, performing heart transplantation or implantation of a mechanical circulatory support (MCS) device should be considered. However, while the role of durable MCS is well established in patients with dilated cardiomyopathy, implantation of an assist device in patients with restrictive physiology and preserved systolic function is rarely utilized. The main limitation in patients with restrictive cardiomyopathy remains a small left ventricular cavity size that usually prevents the standard inflow canula insertion and could result in impaired inflow into a ventricular assist device, development of suction events, and inflow obstruction.^19^ Heart transplantation remains, therefore, the main treatment option for eligible patients with advanced heart failure and restrictive physiology. Notably, extensive pre-transplant workup should be performed to identify any underlying disease, in order to minimize the risk of calcification recurrence in the transplanted heart. Long-term post-transplant follow-up was unremarkable in our patient without any evidence of re-accumulation of myocardial calcifications following heart transplantation. This fact supports the theory that idiopathic myocardial calcifications manifest an intracardiac rather than a systemic process.

In conclusion, massive myocardial calcifications could result in end-stage heart failure. Performing orthotopic heart transplantation in a patient with idiopathic myocardial calcifications leading to end-stage heart failure might be considered a safe and reasonable therapeutic option.

## Lead author biography


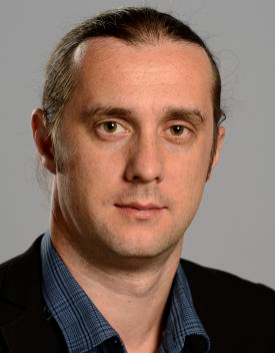


Dr. Alexander Fardman is a senior cardiologist and head of the mechanical circulatory support service in heart transplantation and mechanical circulatory support unit of Sheba Medical Center. He completed a cardiovascular fellowship in Sheba Medical Center and post-graduate course in heart failure (PCHF) in the University Hospital of Zurich (USZ). He completed a clinical observership in advanced heart failure at Deutsches Herzzentrum der Charité (DHZC).

## Data Availability

The data underlying this article will be shared on reasonable request to the corresponding author.
